# Podocyte Autophagy in Homeostasis and Disease

**DOI:** 10.3390/jcm10061184

**Published:** 2021-03-12

**Authors:** Qisheng Lin, Khadija Banu, Zhaohui Ni, Jeremy S. Leventhal, Madhav C. Menon

**Affiliations:** 1 Division of Nephrology, Medicine, Icahn School of Medicine at Mount Sinai, New York, NY 10029, USA; linqisheng@sjtu.edu.cn (Q.L.); khadija.banu@yale.edu (K.B.); jleventhal@wphospital.org (J.S.L.); 2 Department of Nephrology, Renji Hospital, School of Medicine, Shanghai Jiao Tong University, Shanghai 200127, China; nizhaohui@renji.com; 3 Division of Nephrology, Yale School of Medicine, New Haven, CT 06510, USA

**Keywords:** autophagy, podocyte, signaling pathway, homeostasis, glomerular disease

## Abstract

Autophagy is a protective mechanism that removes dysfunctional components and provides nutrition for cells. Podocytes are terminally differentiated specialized epithelial cells that wrap around the capillaries of the glomerular filtration barrier and show high autophagy level at the baseline. Here, we provide an overview of cellular autophagy and its regulation in homeostasis with specific reference to podocytes. We discuss recent data that have focused on the functional role and regulation of autophagy during podocyte injury in experimental and clinical glomerular diseases. A thorough understanding of podocyte autophagy could shed novel insights into podocyte survival mechanisms with injury and offer potential targets for novel therapeutics for glomerular disease.

## 1. Introduction: Autophagy Overview

The word “autophagy” or self-eating was first coined by C de Duve in 1963, to describe the degradation and eventual recycling of cytoplasmic components in the lysosome/vacuole [1]. Autophagy can be broadly divided into macroautophagy, microautophagy, and chaperone-mediated autophagy (CMA) [2]. Macroautophagy is the canonical process where an autophagosome with double-membraned vesicles engulfs damaged or aged organelles and then fuses with lysosomes for degradation. When the lysosomal membrane directly invaginates, encloses, and degrades small cytoplasmic fragments, the process is called microautophagy. In CMA, selected proteins with motifs necessary for binding to a specific heat shock protein (HSPA8) are chaperoned into the lysosomal lumen via lysosomal associated membrane protein 2 (LAMP2) interactions. Among these generic autophagy mechanisms, macroautophagy has been most extensively studied with respect to kidney disease.

Essentially, autophagy is induced by various cellular stressors, including cellular damage, intracellular pathogens, nutrient deprivation and hypoxia, and aims to provide a bioenergetic benefit for cell survival [3]. Autophagy sequesters injured cytoplasmic structures and macromolecules to generate the oxidizable substrates and other molecules, such as amino acids, which are essential for cell survival [4]. Some generated amino acids are further processed to promote the tricarboxylic acid (TCA) cycle, which generates adenosine triphosphate (ATP) for cellular function. It also works as a biosynthetic machine since intermediates in the TCA cycle can be converted into various metabolites, including cytosolic acetyl CoA, sterols, nucleotides, and even glucose [3]. In addition to the cell-autonomous role for autophagy in promoting survival, autophagy pathway proteins are also involved in mitigating programmed cell death or apoptosis [5]. Silencing of autophagy genes beclin−1/BECN1 showed no effect on cell death or pathogen spread, but resulted in uncontrolled programmed cell death beyond sites of pathogen infection in plant cells [5].

Exhaustive and elegant reviews have described the role of autophagy in renal tubular epithelial cells (RTECs) in renal disease [6]. Our review will focus on macroautophagy or autophagy, and its role in renal glomerular epithelial cells or podocytes, both in homeostasis and pathological conditions. We will examine recent data on the regulation and role of podocyte autophagy, with emphasis on the suggested role of autophagy in podocyte survival. We highlight potential platforms for future research regarding autophagy that are relevant to podocyte injury in human disease.

## 2. Regulation of Autophagy Pathway

**Figure 1:**
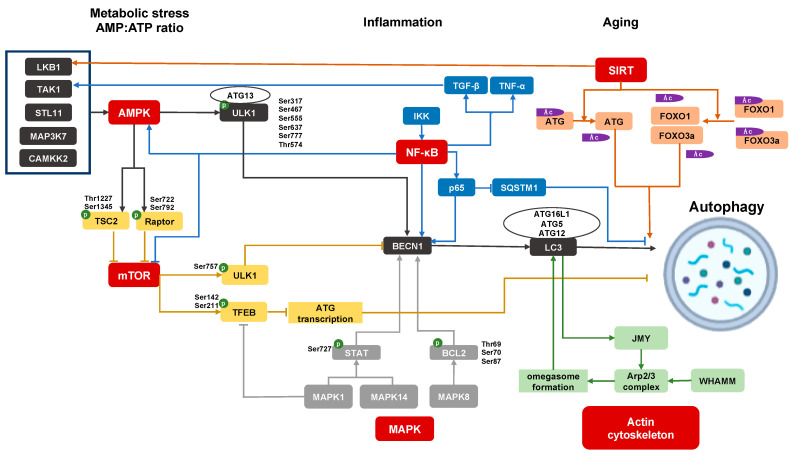
Regulation of autophagy pathway in podocyte. AMP-activated protein kinase (AMPK) signaling, mammalian target of rapamycin (mTOR) signaling, sirtuins (SIRT) signaling, mitogen-activated protein kinases (MAPK) signaling, inflammatory pathways, and actin cytoskeleton pathway participate in podocyte autophagy; p: Phosphorylation; Ac: Acetylation.

The protective roles of autophagy can be summarized as providing energy to maintain homeostasis, as well as cell repair by eliminating defective macromolecules and organelles in cells [7]. The former ensures that autophagy is activated by canonical energy conservation pathways in order to produce nutrients and promote cell survival in starvation stress or increased growth needs. The latter is a quality control function of autophagy to promptly clear, abnormal accumulated proteins and dysfunctional organelles, as well as invading bacteria, to maintain the normal physiological function of cells. Hence, the major regulatory pathways for autophagy reflect these functions. Major regulatory pathways intersect with autophagy via activating or inhibiting major autophagy genes (ATGs). In Figure 1, we summarize the candidate upstream events and signaling cascades regulating the autophagy pathway in podocytes. Notably, autophagy can also be considered as a proverbial double-edged sword depending on the cell- and disease-type in which its role is tested. For instance, excessive activation of autophagy prevented the cell death of tumors (reviewed in [8]), while persistent renal tubular cell autophagy promoted kidney fibrosis and hepatocyte autophagy promoted liver fibrosis, respectively [9,10].

### 2.1. AMPK Signaling

AMP-activated protein kinase (AMPK) signaling is an important stimulus for induction of autophagy. In cellular nutrient deprivation, upstream kinases, such as serine/threonine protein kinase (STK11)/liver kinase B1 (LKB1), calcium/calmodulin­dependent protein kinase 2 (CAMKK2), and mitogen­activated protein kinase 7 (MAP3 K7)/transforming growth factor beta-activated kinase 1 (TAK1), regulate AMPK activation through the modulation of the cytosolic ATP-to-AMP ratio [11,12,13,14,15]. AMPK regulates autophagy via two signals: Inhibition of mammalian target of rapamycin (MTOR) or direct phosphorylation of ULK1. AMPK can phosphorylate TSC2 (tuberous sclerosis complex 2, an upstream regulator of MTOR) on Thr1227 and Ser1345, and RAPTOR (regulatory-associated protein of MTOR, a mTORC1 binding protein) on Ser722 and Ser792 [16,17], which result in the inhibition of MTOR activation, mTORC1 complex formation, and promoting downstream autophagy (discussed below). AMPK also directly phosphorylates ULK1 at Ser317, Ser467, Ser555, Ser637 and Ser777, and Thr574 [18,19]. After phosphorylation, the activated ULK1 recruits FIP200, autophagy-related gene 13 (ATG13), and ATG101 to form the ULK1 complex. Subsequently, ULK1 activates the class III phosphatidylinositol 3-kinase (PI3 K) complex 1, including VPS14, VPS34, BECN1, and ATG14 L. The class III PI3 K complex 1 generates phosphatidylinositol−3-phosphate, which helps shape the phagophore membrane. The recruitment of ATG16 L1 and ATG5-ATG12, catalyzes the covalent attachment of the carboxy-terminal glycine of LC3 to the phagophore membrane forming autophagosomes [20].

### 2.2. MTOR Signaling

MTOR forms two complexes, mTOR complex 1 (mTORC1) and mTORC2. The evolutionarily conserved role of the mTORC1 complex is to promote anabolic metabolism for cell growth and proliferation, and is regulated by cytoplasmic amino acid levels [21]. The mTORC1 regulates ULK1 activation by phosphorylation at Ser757, which is inhibitory to ULK1 activation and the formation of ULK1 and class III PI3 K complexes, and therefore to autophagy [22]. The mTORC1 also phosphorylates transcription factor EB (TFEB) at Ser142 and Ser211, preventing TFEB translocation to the nucleus, and reducing lysosomal and autophagy gene transcription [23,24]. As a result, when mTORC1 signaling is inhibited in conditions of nutrient or growth factor deprivation with low cellular energy levels, autophagy is in turn activated via ULK1 and class III PI3 K complexes, as discussed above.

### 2.3. TOR-Autophagy Spatial Coupling Compartments (TASCCs) and Feedback Regulation of MTOR and Autophagy

Recent data have offered insight into signals connecting the cellular amino acid pool from increased autophagic flux to the feedback dis-inhibition of the MTORC complex. Autolysosomes and mTORC1 complexes were identified as colocalizing in the cytoplasmic compartment at the trans side of the Golgi apparatus, a spatial coupling arrangement called TASCC [25]. Transfection with the GFP-mRFP-LC3 plasmid (where late autophagosomes are signified by red fluorescence, distinct from early autophagosomes) showed that the TASCC region itself was mostly occupied by autolysosomes with a prominent red signal, while mixed red-green signals were found in the isolated puncta surrounding the TASCC [25]. Treatment with lysosomal proteases and/or an amino acid-free medium demonstrated a significant mTOR dispersion from the TASCC. These data indicated that amino acids derived from autolysosome mediated recycling or breakdown, are important to mTOR recruitment at the TASCC [25]. Furthermore, these data report that TASCCs are novel protein synthesis–breakdown complexes within cells where amino acids from degraded cellular proteins are released, coupled with the activation of protein synthesis, depending on the nutrient milieu. Importantly, the presence of TASCCs was identifiable in high autophagy cells including glomerular podocytes where TASCC formation is likely to sequester mTOR, as well as regulate autophagy [25,26]. A recent study demonstrated that TASCCs are also observed in human AKI-CKD transition, and that TASCC formation and autophagy from RTECs undergoing G2-M arrest after acute injury, promoted kidney fibrosis [27].

### 2.4. Sirtuin Signaling

Sirtuins (SIRT), are a family of NAD^+^-dependent class III histone deacetylases involved in metabolic regulation, and currently include proteins SIRT1 to SIRT7 [28]. Under conditions of energy depletion, NAD^+^ levels are increased and activate downstream SIRT1. SIRT1 then mediates the deacetylation of ATG proteins directly, as well as deacetylation of the transcription factors forkhead box protein O1 (FOXO1) and FOXO3, all resulting in the activation of autophagy to restore energy homeostasis and NAD levels [29,30]. Furthermore, SIRT1 can also trigger LKB1 deacetylation activating AMPK to maintain energy metabolism, cell survival, and autophagy [31,32].

### 2.5. MAPK Signaling and BECN1

The mitogen-activated protein kinases (MAPK), a family of serine/threonine-specific protein kinases, can regulate autophagy. MAPK1/p42 MAPK/extracellular regulated protein kinases 2 (ERK2) phosphorylates TFEB to inhibit TFEB nuclear translocation, inhibiting the transcription of autophagy genes under basal conditions [33]. MAPK8/c-Jun *n*-terminal kinases 1 (JNK1) also trigger phosphorylation of B-cell lymphoma 2 (BCL2), at Thr69, Ser70, and Ser87, which disrupts the BCL2/BECN1 complex and promotes autophagy [34]. The MAPK1 and MAPK14/p38 MAPK phosphorylate signal the transducer and activator of transcription 3 (STAT3) at Ser727, regulating autophagy genes including BECN1 [35].

BECN1 is essential for the onset of autophagy, and is a widely-used signal protein to monitor cellular autophagy levels [36,37]. BECN1 is inhibited by binding with anti-apoptotic protein BCL2 through a BH3 protein domain of BECN1 [38]. Autophagy is induced when BECN1 is released from BCL2 by other competing pro-apoptotic BH3 proteins [39]. Meanwhile, direct phosphorylation of BECN1 by death-associated protein kinase 1 (DAPK1) at Thr119, by ULK1 at Ser14, and/or phosphorylation of BCL2 by MAPK8, all activate BECN1 and promote BECN1-induced autophagy via BECN1-associated phosphatidylinositol−3-kinase activity, which is sufficient to initiate autophagosome formation [39,40].

### 2.6. Inflammatory Pathways and Regulation of Autophagy

IκB kinase (IKK) and nuclear factor kappa-light-chain-enhancer of activated B-cells (NF-κB) signaling directly regulate autophagy. The upregulation of IKK/NF-κB signaling pathway, leads to increased nuclear translocation of NF-κB and subsequently promotes binding of the BECN1 promoter, which facilitates the transition from apoptosis to autophagy [41]. P65/RelA, a NF-κB family member, can upregulate BECN1 mRNA and protein levels, and promote autophagy induction [42]. NF-κB induces the delayed accumulation of the autophagy receptor p62/SQSTM1 and promotes autophagosome formation and mitochondrial clearance restricting the inflammatory activity [43]. IKK is also demonstrated to promote the autophagy pathway by NF-κB-independent mechanisms through regulating the impact of mTOR on autophagy, promoting AMPK and JNK signaling pathways [44].

Growth factors, such as transforming growth factor beta (TGF-β) and inflammatory cytokines such as tumor necrosis factor alpha (TNF-α), also enhance TAK1 activation to induce autophagy [45]. TAK1 deficiency exhibits suppressed AMPK activity, increased mTOR activity, and reduced autophagy in response to starvation, which can be rescued by ectopic AMPK activation [46]. TAK1-binding protein 1 (TAB1), TAB2, and TAB3 are three essential co-factors for cytokine-induced activation of the TAK1 complex. The siRNA-based depletion of TAK1 or TAB2, or the inhibitor of TAK1 effectively abrogated AMPK activation, mTORC1 inhibition, and autophagosome formation [14,47,48]. Under normal conditions, TAB2 and TAB3 bind the essential autophagic factor BECN1 at a TAB-binding domain. However, TAB2 and TAB3 dissociate from BECN1 and then engage in the above stimulatory interactions with TAK1 in milieu of nutrient deprivation [49,50]. Both aspects in turn promote autophagy. TAK1, as an upstream regulator of IKK can also regulate autophagy through the IKK/NF-κB signaling pathway [35,49,51,52].

### 2.7. Actin Cytoskeleton and the Regulation of Autophagy

The interactions of the autophagy pathway with the regulation of cellular actin cytoskeletal dynamics are likely to be especially relevant in specialized epithelial cells with intricate cytoskeletal arrangements, as well as high basal autophagy levels, typified by glomerular podocytes. In nutrient deprivation, a cytoskeleton protein, WASP homolog associated with actin, membranes, and microtubules (or WHAMM), is recruited to the endoplasmic reticulum and activates the Arp2/3 complex to form an actin-branched network facilitating the formation of the omegasome [53]. Meanwhile, the phagophore with ATG9-rich membranes can merge to expand the omegasomes, and LC3 II is inserted into the phagophore membrane to trigger autophagy [54]. LC3 II also recruits the junction-mediating and regulatory protein (JMY), another nucleation-promoting factor for the Arp 2/3 complex [55,56]. Hence, the branched actin network induced by WHAMM and JMY is important for expansion of the phagophore membrane and formation of autophagosomes [53]. Finally, the mature autophagosomes that detach from the omegasome, are transported by dynein-dynactin along microtubules, and fused with late endosome and lysosomes for degradation [57]. These fusion events depend on membrane-cytoskeleton adaptors, such as annexins, actin, and myosin I [57]. The interaction of autophagy and cytoskeleton is best brought out by inhibitors of cytoskeleton (cytochalasin B or D that induce the depolymerization of microfilaments), which interfere with the formation of autophagosomes. Hence, cytoskeletal regulatory proteins and cytoskeleton are important to autophagy. The effects of cytoskeleton on autophagy are likely to be regulated by specific adapters in selected cell types since nocodazole, another inhibitor for cytoskeleton by inducing depolymerization of microtubules, showed no effect on autophagy in rat kidney cells [58]. The reported roles of cytoskeletal injury and dysregulation of autophagy in podocytes are discussed in the section below (Section 4.4). It is important to note that only limited data exist regarding the occurrence of dysregulation of cytoskeleton with primary deficiencies of the autophagy deficiency pathway in most cell types including podocytes.

## 3. Renal Epithelial Cell Autophagy in Homeostasis

### 3.1. Autophagy Deficiency during Nephron Development

**Figure 2:**
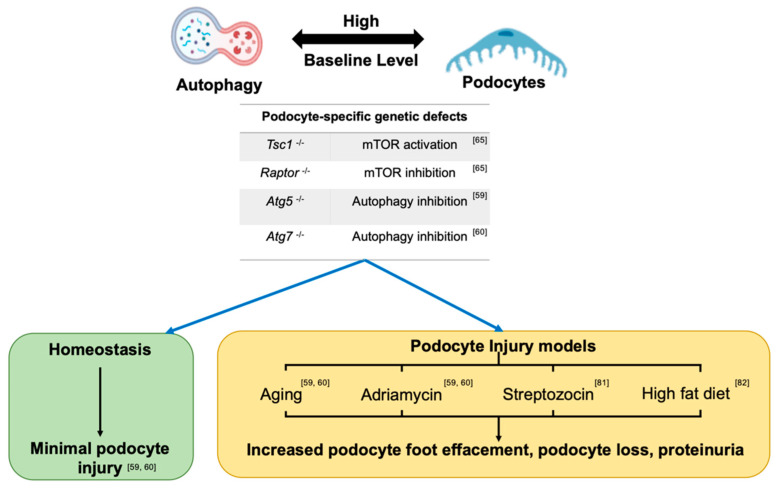
Role of dysregulated autophagy pathway in podocyte biology in homeostasis and injury models. Podocytes have high baseline levels of autophagy. The AMPK signaling pathway was reportedly predominant for autophagy regulation in a recent study evaluating the autophagy level in podocyte-specific knockout *Tsc1* mice and podocyte-specific knockout *Raptor* with an AMPK activator. Autophagy inhibition during podocyte development shows no overt podocyte phenotype until early adulthood in homeostasis. However, the podocyte-specific knockout of *Atg5* or *Atg7*, or disruptions of mTOR signaling in mice aggravated podocyte injury with podocyte foot effacement, podocytopenia, and proteinuria on exposure to aging or injury with adriamycin (ADR), high fat diet or streptozocin injury. These data indicate a protective role for autophagy in podocyte injury.

Figure 2 summarizes important experimental and translational data regarding the role of dysregulated autophagy pathway in podocyte biology in homeostasis and injury models. Data from tissue-specific, single autophagy gene knockout mice, suggest that autophagy deficiency could be more dispensable in podocytes during certain stages in renal development, but plays more important roles in RTEC development [59,60,61,62,63]. For instance, podocyte-specific knockout of *Atg5* [59] or *Atg7* [60] (driven by Nphs2-cre) showed no evidence of proteinuria or histologic injury on a light microscopic examination of adult mice up to 6 months of age. Proteinuria and histologic glomerular injury occurred in these *Atg5* knockout mice only with at least 12 months of ageing. Notably, in this report, the EM examination in 4-month-old mice did show evidence of cytoskeletal disarray or foot process effacement (FPE), indicating a role for autophagy deficiency from *Atg5* knockout mice in the regulation of podocyte cytoskeleton that was evident at least on an ultra-structural examination. In embryonic RTECs, no obvious histological injury was evident in *Atg5* knockout mice at E19.5 [61]. However, the inducible and time-specific deletion of *Atg5* in RTECs (*Atg5*^flox/flox^; *Pax8.rtTA; tetO.Cre*) resulted in ultrastructural alterations and increased serum creatinine after doxycycline administration for 1 and 5 months [61]. Proximal RTEC-specific autophagy deficient, *Atg7^flox/flox^; KAP-Cre^+^* mice revealed higher levels of KIM−1 (a biomarker of RTEC injury and AKI), more TUNEL-positive apoptotic RTECs, and increased presence of intracellular inclusions and abnormal structures in 2-month old knockout mice versus controls. These injury phenotypes were also aggravated in an age-dependent manner [62]. Contrastingly, the pan-nephron progenitor epithelial cell knockout of *Atg5* (*Six2-Cre; Atg5^flox/flox^*, including podocytes, parietal epithelial cells, proximal tubule, loop of Henle, and distal tubule) revealed much more severe glomerular and tubular injury than either podocyte- or RTEC-specific knockout *Atg5* [63]. *Six2-Cre; Atg5^flox/flox^* mice developed podocytes as well as tubular dysfunction within 2 months, with progressive renal dysfunction similar to the human chronic kidney disease by 4 months, and end stage renal failure by 6 months. Similarly, *Six2-Cre; Atg7^flox/flox^* also developed focal segmental glomerular sclerosis (FSGS) and tubular injury by 4 months with moderate albuminuria [63]. These data may also suggest an essential role for the autophagy pathway in early renal epithelial cell development when Six 2 expression is initiated at E10.5 at the stages of ureteric bud outgrowth and metanephric mesenchyme [64].

### 3.2. Baseline Autophagy in Adult Podocytes

Podocytes have demonstrably high basal levels of autophagy from multiple studies. In GFP-LC3 transgenic mice, increased levels of GFP-LC3 positive puncta by immunofluorescence microscopy and more autophagosomes by electron microscopy (EM) were observed in podocytes than in RTECs or other resident kidney cells in homeostasis [59]. Since cytoplasmic autophagosomes could accumulate secondary to impaired degradation or increased autophagy or both, Hartleben et al. evaluated autophagosomes in vitro before-and-after treatment with the lysosomal inhibitor chloroquine, confirming that podocytes exhibited high basal levels of autophagy [59]. Recent exciting work has suggested that the high autophagy levels observed in podocytes at baseline are regulated primarily by AMPK in the longer term, rather than mTOR signaling [65]. Podocyte-specific *Tsc1* or *Raptor* gene deletions, to respectively activate or inhibit mTORC1 signaling, revealed insignificant sustained changes in autophagy in podocytes in vivo. On the other hand, AICAR, an AMPK agonist, upregulated autophagy in wildtype and podocyte-specific knockout *Raptor* mice, indicating that the AMPK-mediated regulation of autophagy is central to the maintenance of high autophagy levels in podocytes in vivo, and is relatively independent from the mTOR signaling pathway [65].

### 3.3. Baseline Autophagy in Renal Tubular Epithelial Cells (RTEC)

In RTECs, baseline autophagy levels may be lower than observed in podocytes in vivo [59]. As mentioned above, GFP-LC3 transgenic mice showed less LC3-puncta and autophagosomes in RTECs than podocytes in homeostasis. Developmental data, however, suggest that RTEC-specific knockout *Atg5* or *Atg7* resulted in the tubular injury [62,63,64]. Hence, the role of autophagy in highly metabolically active RTECs is likely complex. A recent elegant review has described the roles of autophagy in RTECs in development, injury models and its relevance to human disease [6]. 

## 4. Autophagy in Podocyte Injury and Disease

### 4.1. Autophagy in Podocyte Cytoskeletal Change

Podocytes are architecturally specialized cells located at the urinary aspect of the glomerular filtration barrier, and cytoskeletal organization is essential for the regulation of cell morphology and function [66]. Disruption of the podocyte cytoskeleton, seen electron microscopically as FPE, causes proteinuria and can subsequently associate with podocyte loss [67]. Podocytes are incapable of self-renewal, and podocyte loss over a critical threshold is associated with progressive renal failure. As discussed in Section 2.7, actin dynamics and membrane-cytoskeleton scaffolds are known to regulate autophagy in diverse cell types [57]. In podocyte injury, several studies have also observed important inter-correlations between the disrupted autophagy pathway and cytoskeleton. In tunicamycin-induced endoplasmic reticulum (ER) stress in podocytes, both autophagy markers and cytoskeletal protein levels were upregulated. In this context, specific inhibition of autophagy further increased the expression and disordered distribution of the cytoskeletal proteins in podocytes [68]. In a passive Heymann nephritis rat model, ER stress and cytoskeletal injury were again associated with autophagy activation. Silencing *ATG7* aggravated cytoskeletal injury under conditions of ER stress in vitro [69]. Inhibitors of autophagy, such as 3-MA or chloroquine, resulted in aggravated puromycin aminonucleoside-induced nephropathy (PAN), increased podocyte apoptosis, and disruption of the podocyte cytoskeleton seen as FPE [70]. Knockdown of prorenin receptor (PRR), a highly expressed marker in podocytes, blocked autophagosome-lysosome fusion and also altered the cytoskeleton, similar to the role of bafilomycin A1, showing the simultaneous impact of injury on podocyte autophagy and cytoskeleton [71]. As described above, the EM examination of podocyte-specific *Atg5* knockout mice at 4 months of age showed inhibited autophagy and evidence of cytoskeletal disarray, i.e., FPE, suggesting that primary deficiencies of the podocyte autophagy pathway resulted in alterations of cytoskeleton in this murine model. Hence, existing data suggest that podocyte cytoskeletal injury interferes with podocyte autophagy, while inhibited autophagy in the context of injury stimuli, aggravates podocyte loss. However, the role of autophagy in regulating podocyte cytoskeleton and conversely, the regulation of autophagy when cytoskeleton is primarily disrupted, both are aspects that need specific examination.

### 4.2. Podocyte Autophagy in Nephrotic Syndromes: FSGS and MCD

The majority of the evidence from genetic data has suggested that while autophagy deficiency may be partly dispensable during selective stages of podocyte development, exposure to podocyte injury stimuli in adult animals with autophagy deficiency exaggerates injury and promotes podocyte loss [59,60]. When proteinuria results after glomerular injury of diverse etiology, the ultrastructural examination of podocytes in vivo usually demonstrate increased microvillous transformation and protein reabsorption, a likely compensatory phenomenon to protein in the filtration space [59,60]. Conjecturally, in this milieu, the upregulated autophagy pathway (coupled with TASCC formation) may be a compensatory response within podocytes [25,26]. Additionally, as described in Section 2.7, direct perturbations of the podocyte cytoskeleton may themselves alter podocyte autophagy responses [57]. For instance, in both cultured podocyte cell lines and in adriamycin-induced injury (ADR) models in vivo, autophagy is demonstrably upregulated by the ADR treatment [59,60]. Treatment with the autophagy agonist rapamycin, mitigated ADR-induced apoptosis, while chloroquine, an autophagy inhibitor, aggravated ADR-induced podocyte apoptosis [59,60]. Analogously, in vivo, podocyte loss, glomerulosclerosis, and proteinuria were increased in podocyte-specific knockout *Atg7* or *Atg5* mice than control mice upon the ADR treatment [59,60]. Puromycin amino nucleoside induces a minimal change disease (MCD)-like injury in rats at early stages characterized by induction of proteinuria and FPE without podocyte loss (also called PAN) [72]. In PAN models, the administration of autophagy inhibitors, such as 3-MA, resulted in aggravation of proteinuria, extensive FPE, and reduction of podocyte markers, indicating that inhibition of podocyte autophagy in the context of injury stimuli promoted the conversion of an MCD-phenotype (with maintained podocyte numbers) to podocytopenia and FSGS development [72]. Other animal data also suggest that maintenance of an optimal level of mTOR signaling in podocytes, and consequently maintaining autophagy, is important to podocyte survival, since both mTOR hyperactivation models or pharmacological mTOR inhibition led to aggravated podocyte loss and FSGS [73]. Most interestingly, from human data, podocytes in biopsies of patients with MCD showed significantly increased levels of LC3-II by immunoblots and increased double-membrane bound vacuoles (consistent with autophagosomes) by electron microscopy compared to FSGS biopsies [72]. Furthermore, repeat renal biopsies in this study demonstrated that MCD patient biopsies where high podocyte autophagic activity was persistent, retained the MCD phenotype without podocyte loss, whereas patients with reduced podocyte autophagic activity showed progression to FSGS [72]. These translational data may suggest that the compensatory upregulation of autophagy may promote podocyte survival when cytoskeletal dysregulation occurs and is accompanied by proteinuria, helping to prevent progression to FSGS in these settings. In this context, inhibition of autophagy could transition to early podocyte loss and FSGS. These data are highly relevant to human disease with potential for novel therapeutics in the context of podocyte injury. In Table 1, we list some of the reported autophagy activators and inhibitors, as well as their in vitro and in vivo effects on podocytes in homeostasis and injury models.

### 4.3. Diabetic Kidney Disease

Several diabetic kidney disease (DKD) studies have focused on autophagy in diabetic podocytes. In vitro, high glucose-treated podocytes show upregulated autophagy at 48 h, indicating that hyperglycemia activates podocyte autophagy in early stages. Interestingly, however, autophagy was reduced after high glucose for longer time periods (15 days) [81]. Similarly, streptozocin (STZ)-treated diabetic mice show upregulation of autophagy in glomeruli and podocyte at 1 and 4 weeks (before the development of significant overt diabetic kidney disease). However, downregulation of podocyte autophagy was seen by 8 weeks [81]. The kidney-to-body weight ratio, BUN levels, and podocyte loss were more severe in STZ-treated podocyte-specific *Atg5* knockout mice than control mice, suggesting aggravated diabetic kidney disease with STZ by podocyte specific autophagy deficiency [81]. In Otsuka Long-Evans Tokushima fatty (OLETF) rats, an obesity­induced type-II diabetes rat model, high fat diet also induced inhibition of autophagy in podocytes [82]. Here again, compared to control mice, podocyte-specific autophagy deficient mice (*Nphs2-Cre; Atg5^flox/flox^*) with high-fat diet developed worsened albuminuria accompanied by more severe podocyte damage [82]. In this set of experiments, serum from OLETF rats on a high-fat diet or podocyte-specific autophagy deficient mice with diabetic kidney disease patients were then added to the media for podocyte culture to wildtype podocytes. Podocytes cultured with these sera displayed autophago-lysosome dysfunction and increased apoptosis [82]. Kidney biopsy samples from diabetic kidney disease patients with massive proteinuria have also shown reduced autophagy levels in podocytes [82]. Other data suggest that lysosome dysfunction in cells in a diabetic milieu may inhibit the degradation of autophagosomes, resulting in impaired autophagy in diabetic kidney disease patients [83]. From human clinical trials testing the oral hypoglycemic agent, metformin (an AMPK and therefore, autophagy activator), significant benefits have been observed in delaying the onset and severity of proteinuria with diabetic kidney disease [84]. Together, these data suggest that autophagy is protective in podocytes against high glucose injury in a diabetic milieu, while prolonged hyperglycemia may itself downregulate autophagy contributing to the progression of diabetic kidney disease. Therefore, in diabetes and diabetic kidney disease, activation of podocyte autophagy could offer a therapeutic benefit.

### 4.4. Lupus Nephritis

With reference to models of lupus nephritis (LN), autophagy is upregulated in MRL^lpr/lpr^ mice, LN patients, and in podocytes treated with IFN-α and sera from patients with LN [85]. Further mechanistic data show that autophagy induced by IgG extracted from LN patients could play a protective role in podocyte apoptosis, and inhibition of autophagy by 3-MA and ATG5 siRNA aggravate podocyte damage [85]. Importantly, in inflammatory nephritides such as LN, autophagy plays important and complex roles in T-cells [86], as well as B-cells and antibody production [87,88], the discussion of which are beyond the scope of our current review. Independent roles of autophagy in immune cells, as well as renal epithelial cells will influence the outcomes and any potential autophagy-based therapeutics in glomerulonephritis. 

## 5. Conclusions

In summary, we review pathways regulating cellular autophagy, and highlight recent data on the regulation of podocyte autophagy in hemostasis and disease. Several data suggest that the lack of autophagy aggravates podocyte injury in experimental disease models. Data from human diseases with podocyte injury such as MCD, FSGS, DKD, and LN, also suggest a protective role for autophagy pathway in podocytes. Whether the reliance of podocytes on increased autophagy during injury is a consequence of the generic disruption of their complex cytoskeleton in disease models, protein reabsorption mechanisms or specific to the type of podocyte injury, requires further investigation. Furthermore, downstream mechanisms involved in pro-survival effects once podocyte autophagy is activated including favorable bioenergetics, direct cytoskeletal regulation or cell volume regulation all need to be examined in future work. A generic protective role for autophagy in podocyte cytoskeletal injury would be of considerable therapeutic interest in human glomerular disease, and serve as an exciting platform for podocyte research.

## Figures and Tables

**Table 1:** Inhibitors and activators of podocyte autophagy.

Drug	Autophagy	Podocyte Type	Condition	Pharmacological Effect	Ref
Chloroquine (CQ)	Inhibitor	human cell line	PAN ^1^	Decrease cell motility, disrupt actin cytoskeleton	[74]
		mouse cell line	Bbf ^2^	Increase podocyte injury and Bbf cytotoxicity	[75]
3-MA	Inhibitor	mouse cell line	Ang II ^4^	Increase apoptosis	[76]
		mouse cell line	LPS ^5^	Aggravate podocyte injury and ER stress	[77]
		mouse cell line	HG ^6^	Increase apoptosis	[78]
		rat	PAN	Earlier onset and greater proteinuria, more extensive FPE ^3^, reduction in podocyte markers	[72]
Rapamycin	Activator	mouse cell line	Bbf	Reduce podocyte injury and Bbf cytotoxicity	[75]
		mouse cell line	LPS	Alleviate podocyte injury and ER stress	[77]
		mouse	STZ ^7^	Alleviate FPE, glomerular basement membrane thickening, and matrix accumulation	[79]
		rat	PAN	Decrease proteinuria and severe FPE	[72]
Valproate (VPA)	Activator	rat	STZ	Improve renal function, decrease apoptosis and DNA damage, ameliorate the histological alterations and FPE	[80]

^1^ PAN: Puromycin aminonucleoside. ^2^ Bbf: Benzo [b] fluoranthene. ^3^ FPE: Foot-process effacement. ^4^ Ang II: Angiotensin II. ^5^ LPS: Lipopolysaccharide. ^6^ HG: High glucose. ^7^ STZ: Streptozocin.

## Data Availability

No new data were created or analyzed in this study. Data sharing is not applicable to this article.
